# Temporal dynamics of blood pressure, functional status and cognitive function in adults aged 85 years and older: a dynamic time warping approach in the Leiden 85-plus study

**DOI:** 10.1093/ageing/afag006

**Published:** 2026-01-29

**Authors:** Jan J Duin, Stella Trompet, Erik Giltay, Jacob T Johnson, Jacobijn Gussekloo, Rosalinde R K E Poortvliet, Simon P Mooijaart, Frederiek van den Bos

**Affiliations:** Department of Internal Medicine, Section of Gerontology and Geriatrics, Leiden University Medical Center, Leiden, The Netherlands; LUMC Center for Medicine for Older People, Leiden University Medical Center, Leiden, The Netherlands; Department of Internal Medicine, Section of Gerontology and Geriatrics, Leiden University Medical Center, Leiden, The Netherlands; LUMC Center for Medicine for Older People, Leiden University Medical Center, Leiden, The Netherlands; Department of Psychiatry, Leiden University Medical Center, Leiden, The Netherlands; Health Campus The Hague, Department of Public Health and Primary Care, Leiden University Medical Center, Leiden, The Netherlands; Department of Internal Medicine, Section of Gerontology and Geriatrics, Leiden University Medical Center, Leiden, The Netherlands; LUMC Center for Medicine for Older People, Leiden University Medical Center, Leiden, The Netherlands; Department of Internal Medicine, Section of Gerontology and Geriatrics, Leiden University Medical Center, Leiden, The Netherlands; LUMC Center for Medicine for Older People, Leiden University Medical Center, Leiden, The Netherlands; Department of Public Health and Primary Care, Leiden University Medical Center, Leiden, The Netherlands; LUMC Center for Medicine for Older People, Leiden University Medical Center, Leiden, The Netherlands; Department of Public Health and Primary Care, Leiden University Medical Center, Leiden, The Netherlands; Department of Internal Medicine, Section of Gerontology and Geriatrics, Leiden University Medical Center, Leiden, The Netherlands; LUMC Center for Medicine for Older People, Leiden University Medical Center, Leiden, The Netherlands; Department of Internal Medicine, Section of Gerontology and Geriatrics, Leiden University Medical Center, Leiden, The Netherlands; LUMC Center for Medicine for Older People, Leiden University Medical Center, Leiden, The Netherlands

**Keywords:** dynamic time warping, blood pressure, cognitive function, functional status, geriatrics, older adult, octogenarians

## Abstract

**Introduction:**

High blood pressure (BP), functional decline, and cognitive decline commonly co-occur in late life. The temporal dynamics between changes in BP and changes in functional or cognitive status remain unclear. The aim of this study is to explore these temporal dynamics in adults aged 85 years and older using dynamic time warping (DTW).

**Methods:**

This study used data from the Leiden 85-plus Study. BP, functional status and cognitive function were measured at baseline (age 85) and annually over 5 years. Participants with at least three measurements were included (*n* = 429 of 599). Functional status was assessed using the Groningen Activity Restriction Scale for Activities of Daily Living (ADL) and Instrumental Activities of Daily Living (IADL). Cognitive function was measured with the Mini-Mental State Examination (MMSE). DTW was applied to assess temporal relationships.

**Results:**

The 429 participants were all aged 85 years at baseline, 69% were female and 39% used antihypertensive medication. Increases in BP over time were associated with later IADL decline, while BP decreases were associated with later IADL improvement (*P* < .05). Similarly, decreases in diastolic BP over time were associated with later decreases in MMSE, while increases in diastolic BP were linked to later improvements in MMSE (*P* < .05).

**Conclusion:**

BP changes were associated with subsequent inverse IADL changes over time, while changes in diastolic BP preceded concordant changes in cognitive function. These findings highlight the importance of BP changes in old age, as they might indicate later functional or cognitive decline.

## Key points

- Blood pressure changes precede functional decline in adults aged 85+.
- Rising blood pressure leads to worsening functional status.
- Falling diastolic pressure predicts later cognitive decline.
- Blood pressure stability matters more than absolute values in old age.
- Dynamic time warping reveals hidden temporal patterns in ageing data.

## Introduction

With ageing comes an increased prevalence of high blood pressure (BP), which has a prevalence of >70% in adults aged 80 years and older [1]. High BP in midlife is strongly associated with functional and cognitive decline in late life [2, 3], but the relationship between BP measured in late life and functional status and cognitive function appears more complex. Evidence suggests that in certain older populations, high BP might even be protective against functional and cognitive decline [4–6].

Given this complexity, and the relative paucity of dedicated studies assessing this relationship, the management of BP in older adults remains a topic of ongoing debate. Evidence from the Hypertension in the Very Elderly Trial (HYVET) supports the potential benefits of intensive BP control in older adults [7]. In contrast, observational studies have reported that lower BP levels may be associated with adverse outcomes, particularly in individuals with frailty or multiple comorbidities [8–10]. These conflicting findings, likely influenced by differences in study design, populations and methodological approaches, highlight the need for a more nuanced understanding of how BP relates to functional status and cognitive function in old age, particularly by taking into account the dynamic changes within individuals over time.

Most prior research has relied on cross-sectional or longitudinal designs using linear models to assess the associations between BP, functional status and cognitive function over time [4–6, 8, 11, 12]. While some studies have explored non-linear approaches [13–15], these methods, whether linear or non-linear, still examine group-level averages that may obscure individual-level BP changes that occur at different times within the sample. Dynamic time warping (DTW), a network-based method for analysing time-series data, offers a possible alternative by identifying temporal patterns that might otherwise be overlooked [16–18]. DTW first captures temporal relationships within individuals before aggregating patterns across the group. Although DTW has been applied in psychiatry [16, 19, 20], it remains underutilised in the study of somatic diseases, specifically in geriatric research. This study therefore serves as a proof of principle for applying DTW to somatic time-series data in geriatric research.

By applying DTW, this study aims to investigate the temporal dynamics between changes in BP and changes in functional status and cognitive function in adults aged 85 years and older.

## Methods

### Leiden 85-plus study

The Leiden 85-plus Study is a prospective, population-based cohort study designed to investigate the health and function of 85-year-olds over a 5-year follow-up period. Comprehensive details regarding the study design, methodology and strategies to address missing data are available in prior publications [21, 22]. In brief, all residents of Leiden, The Netherlands, who reached the age of 85 between 1 September 1997 and 1 September 1999, were invited to participate. There were no additional selection criteria, ensuring a representative sample of the population. Subjects were visited at their home within one month after their 85th birthday for face-to-face interviews, physical and psychological testing. Subjects were revisited annually until the age of 90 years. Ethical approval was obtained from the Medical Ethics Committee of Leiden University Medical Center (approval code P1.150) and all participants provided informed consent. For participants with severe cognitive impairments, informed consent was obtained from legal guardians.

For the current analysis, participants were included if they completed at least three measurements. Participants with missing data on BP, activities of daily living (ADL), instrumental activities of daily living (IADL), and Mini-Mental State Examination (MMSE) were excluded. No formal sample size calculation was performed for this study. To our knowledge, there are no prior studies applying DTW with these specific measurements, which means that no reliable effect size estimates are available to base a power calculation on. The current work should therefore be viewed as an exploratory, proof-of-principle study.

### Blood pressure measurements

BP was measured annually up to age 90 using a manual sphygmomanometer following a standardised protocol to reduce measurement bias. Measurements were taken twice, and the average was used to minimise measurement variability. BP was recorded after at least 5 minutes of seated rest, with participants avoiding vigorous exercise for at least 30 minutes prior. Systolic blood pressure (SBP) and diastolic blood pressure (DBP) were measured at Korotkoff phases 1 and 5, respectively. Mean arterial pressure (MAP: ⅓ SBP + ⅔ DBP) and pulse pressure (PP: SBP—DBP) were derived.

### Functional status and cognitive function

Functional status and cognitive assessments were performed by trained assessors using validated instruments. Functional status was assessed annually using the Groningen Activity Restriction Scale, a validated instrument for measuring disability in ADL and IADL [23]. Each domain consisted of nine questions with four response options per question, resulting in scores ranging from 9 (no disability) to 36 (maximum disability). For this analysis, ADL and IADL scores were inverted for ease of interpretation (i.e. higher score being indicative of better functional status). Cognitive function was assessed annually using the MMSE, a widely used tool for cognitive screening [24]. Scores range from 0 to 30, with higher scores indicating better cognitive function.

### Sociodemographic and clinical characteristics

Sociodemographic characteristics (including gender and education level) and clinical characteristics (including medical history, medication use and grip strength) were collected during the baseline interview at age 85. Education level was dichotomised as primary education or less versus more than primary education. Smoking habits and alcohol intake were assessed through participant interviews, and antihypertensive medication use was obtained from pharmacy records. Grip strength was measured using a Jamar hand dynamometer (Sammons Preston Inc., Bolingbrook, IL). Each participant completed one practice trial, followed by three test measurements, with the highest value used for analysis.

### Statistical analysis

Sociodemographic and clinical variables at baseline were summarised using descriptive statistics, including means and standard deviations (SD) for normally distributed continuous variables, medians with interquartile ranges (IQR) for skewed continuous variables, and frequencies with percentages for categorical variables, as appropriate.

Before conducting the analyses, the items (SBP, DBP, MAP, PP, ADL, IADL and MMSE) were adjusted. MMSE scores were log-transformed (i.e. log_e_(31-MMSE)) to approach normality, while ADL and IADL scores were adjusted to account for their minimum values before applying log_e_ transformation. All items were subsequently z-standardised to facilitate comparability across scales and centralised to yield the relative changes within participants.

Longitudinal trends of the items were analysed using linear mixed-effects models with random intercepts for participants. To examine the overall correlation between the items, a correlation matrix was computed and visualised using hierarchical clustering.

DTW was employed to analyse the temporal relationships between BP and measures of functional status and cognitive function. DTW is a method for analysing time-series data that can identify temporal relationships between variables. Traditional analyses compare variables measured at the same time points. In contrast, DTW can detect when changes in one variable precede changes in another variable, even if the timing is not perfectly aligned across individuals (Supplementary Figure S1). This approach allows us to identify which changes tend to come first and which follow later [25].

We applied directed analyses to examine temporal relationships to determine which changes in variables preceded changes in others, similarly to the methodology in earlier DTW analyses [16–18]. To ensure biologically plausible temporal associations, the alignment was restricted to a time window of one time point (i.e. the current and next measurement). The ‘symmetric2’ step pattern was used, to enable distance measures to be normalised to the number of assessments (i.e. 3, 4, 5 or 6) within each individual [25]. We applied DTW to compute pairwise directed distances between measures of BP (SBP, DBP, MAP and PP), and functional status and cognitive function (ADL, IADL and MMSE) across all participants. This resulted in two asymmetric directed distance matrices per participant [16, 19, 20]. To construct the group-level directed distance matrix, we performed independent t-tests to determine whether the average directed distances between variable pairs were significantly >0 (*P* < .05). To account for multiple testing, we applied the Benjamini-Hochberg procedure to control the false discovery rate at 5% [26]. This correction was applied to all pairwise directed relationships tested.

The group-level directed distance matrix was used to visualise the directed network, where nodes represent variables (e.g. SBP, IADL and MMSE), and arrows indicate temporal relationships at the group level. Green arrows indicate temporal relationships in the same direction, while red indicate changes in opposite directions. For example, a green arrow pointing from the DBP node to the MMSE node would indicate that a decrease in DBP at a given time point is associated with a subsequent decrease in MMSE, or alternatively that an increase in DBP at a given time point is associated with a subsequent increase in MMSE. Conversely, a red arrow pointing from the SBP node to the IADL node would indicate that an increase in SBP at a given time point is associated with a subsequent decrease in ADL, or alternatively that a decrease in SBP at a given time is associated with a subsequent increase in IADL. The strength of the temporal effect is visually represented by the line width of edges (i.e. larger effects result in thicker connections).

Additionally, we calculated in- and outstrength centrality (i.e. temporal lag and temporal lead) to assess the directional influence of each variable. Variables with high outstrength tend to precede changes in multiple other variables, indicated by stronger and more numerous outgoing arrows to all other nodes. Conversely, changes in variables with high instrength are often preceded by changes in other variables, indicated by stronger and more numerous incoming arrows.

To examine potential effect modifications and to ensure the robustness of our findings, sensitivity analyses of the directed DTW analysis were conducted by stratifying participants based on sex (male vs. female), grip strength (median or higher vs. below median), antihypertensive use at baseline (yes vs. no), and changes in antihypertensive medication use during follow-up (no change vs. change). Given that frailty may influence the relationship between BP, functional status, and cognitive function, we used grip strength as a proxy measure for frailty, as comprehensive frailty assessments were not available in the original dataset. Antihypertensive use and changes in antihypertensive use were considered because medication can alter BP dynamics and potentially modify its association with functional status. Additionally, medication adjustments might reflect health deterioration and influence the observed associations.

Only participants without missing outcome data were included in the analysis. Participants that left follow-up before the end of the study were analysed based with the data available up to that point.

Descriptive analyses were performed using SPSS version 25 (IBM Corp Released 2017, IBM SPSS Statistics for Windows, Version 25). All other analyses were performed in R (version 4.2.2) [27]; using the main packages of ‘dtw’ (version 1.23–1) [25] and ‘qgraph’ (version 1.9.8) [28].

## Results

### Patient characteristics

**Figure 1:**
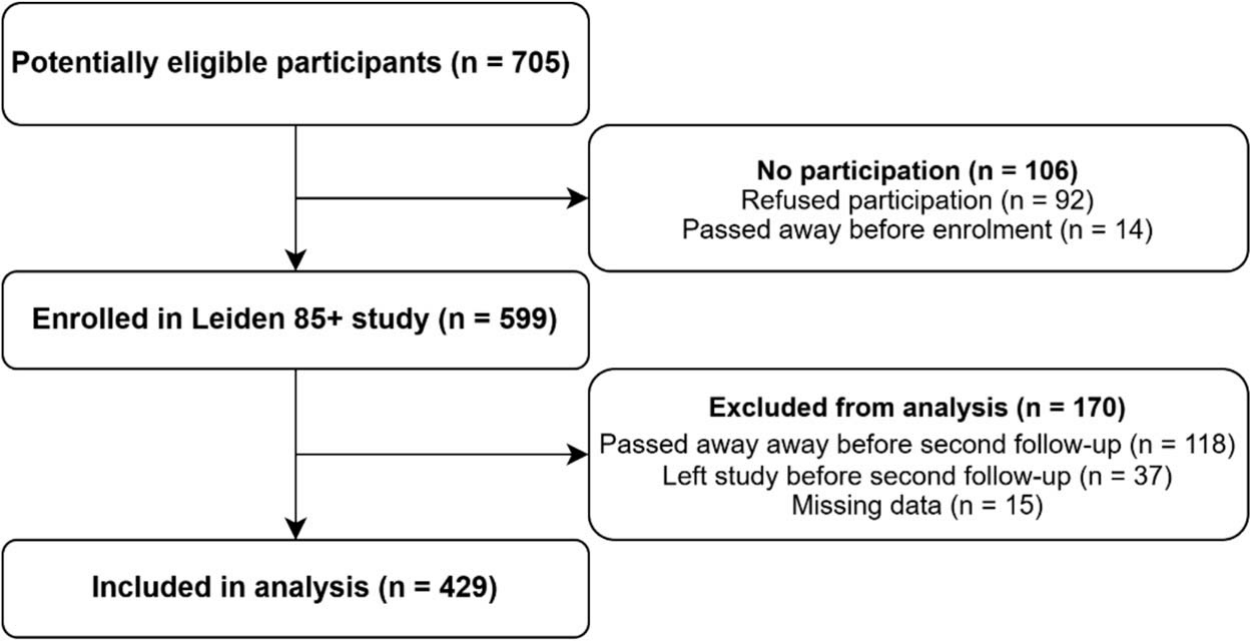
Flow diagram of participant selection for this analysis.

Of the 705 eligible individuals, 599 (85%) provided informed consent and were enrolled in the study. After excluding participants who passed away (*n* = 118) or left the study before the second follow-up (*n* = 37), and those with any missing data on BP, ADL, IADL and MMSE (*n* = 15), the final analytic sample consisted of 429 participants (72% of the original cohort; Figure 1). Out of these, 269 (63% of the included sample) were included up to the final measurement at age 90. Median follow-up time for the analytic sample was 5 years (interquartile range 3–5 years). A detailed overview of follow-up is provided in Supplementary Figure S2.

Table 1 shows the sociodemographic and clinical characteristics of the 429 participants, all aged 85 years at baseline, of whom 296 (69%) were female. At baseline, 166 participants (39%) used antihypertensive medication. Mean (SD) SBP and DBP were 156 (18) mmHg and 78 (9) mmHg, respectively. The median ADL score was 10 (IQR: 9–13), the median IADL score was 17 (IQR: 12–24), and the median MMSE score was 26 (IQR: 24–28). Compared to excluded participants, those included in the analysis were more often female, had higher education levels and were less frequently institutionalised. They also showed higher baseline BP, better functional status and better cognitive function. Detailed comparisons are provided in Supplementary Table S1.

**Table 1:** Baseline characteristics of study participants (aged 85 years)

Characteristic	Study sample (n = 429)
**Demographics**	
Female, n (%)	296 (69)
≤ Primary education, n (%)	264 (62)
Institutionalised, n (%)	62 (15)
**Lifestyle factors**	
Ever smoker, n (%)	194 (45)
Regular alcohol use, n (%)	211 (49)
**Medical history**	
Stroke, n (%)	40 (9)
Myocardial infarction, n (%)	38 (9)
Diabetes mellitus, n (%)	58 (14)
Parkinson’s disease, n (%)	10 (2)
**Medication Use**	
Antihypertensive medication, n (%)	166 (39)
**Blood pressure measures**	
Systolic blood pressure, mmHg, mean (SD)	156 (18)
Diastolic blood pressure, mmHg, mean (SD)	78 (9)
Mean arterial pressure, mmHg, mean (SD)	104 (11)
Pulse pressure, mmHg, mean (SD)	79 (15)
**Functional and cognitive status**	
ADL, median (IQR)	10 (9–13)
IADL, median (IQR)	17 (12–24)
MMSE, median (IQR)	26 (24–28)

Abbreviations: SD = Standard deviation; ADL = activities of daily living; IADL = instrumental activities of daily living; MMSE = Mini-Mental State Examination; IQR = Interquartile range.

### Longitudinal trends

**Figure 2:**
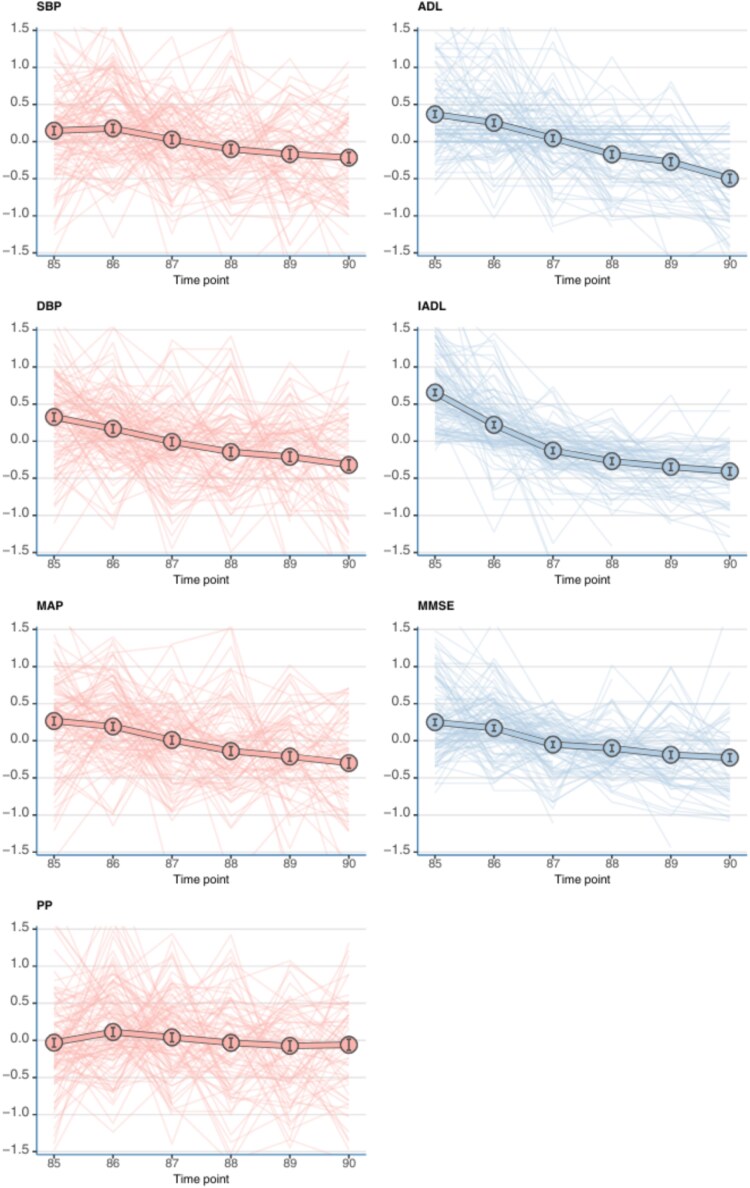
Estimated marginal means (EMMs) over time for systolic blood pressure (SBP), diastolic blood pressure (DBP), mean arterial pressure (MAP), pulse pressure (PP), Activities of Daily Living (ADL), Instrumental Activities of Daily Living (IADL), and Mini-Mental State Examination (MMSE). Values are presented as standardised scores (Z-scores) to allow comparison across domains with different units. Solid lines represent the EMMs at each time point; lighter surrounding lines represent 100 randomly selected individual trajectories, which form the basis for the DTW analyses. ADL and IADL scores were inverted for interpretability, so that higher scores indicate better functional status.

Figure **2** illustrates the longitudinal trajectories of SBP, DBP, MAP, PP, ADL, IADL and MMSE between the ages of 85 and 90. These estimates reflect within-person changes over time, based on linear mixed-effects models using the standardised and centralised scores. Individual trajectories for a random subset of 100 participants are also displayed as these individual time series formed the basis for the subsequent DTW analyses. The average annual changes in all measures are presented in Table 2. All measures showed statistically significant decline over time. A correlation analysis (Supplementary Figure S3) showed that BP parameters clustered together, as did functional and cognitive measures. Correlations between these domains were weak.

**Table 2:** Annual changes in blood pressure, functional status, and cognitive function from age 85 to 90 years

**Variable**	**Annual change (SD/year)**	**95% CI**	***P*-value**
**Blood pressure measures**			
Systolic blood pressure	−0.09	−0.10 to −0.07	<.001
Diastolic blood pressure	−0.13	−0.15 to −0.12	<.001
Mean arterial pressure	−0.12	−0.14 to −0.11	<.001
Pulse pressure	−0.02	−0.04 to −0.01	.008
**Functional and cognitive measures**			
Activities of daily living	−0.18	−0.19 to −0.16	<.001
Instrumental activities of daily living	−0.22	−0.23 to −0.20	<.001
Mini-Mental State Examination	−0.10	−0.12 to −0.09	<.001

Annual changes are expressed as standard deviation units per year based on z-standardised and within-person centralised scores. Higher scores indicate better function for all measures (activities of daily living and instrumental activities of daily living scores were inverted). Abbreviations: SD = Standard deviation; CI = Confidence interval.

### Directed analysis

**Figure 3:**
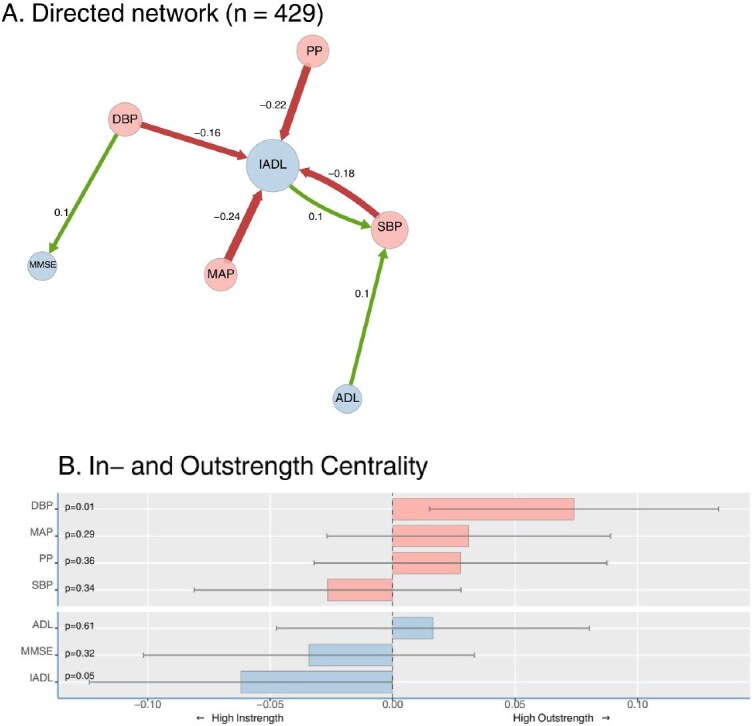
Directed dynamic time warping (DTW) analysis of temporal relationships (A) Directed analysis of time-lagged relationships between blood pressure parameters (SBP, DBP, MAP, PP) and functional measures (ADL, IADL and MMSE) using DTW. Arrows indicate significant time-lagged associations (*P* < .05), where change in one variable (i.e. arrow tail) preceded change in another (i.e. arrow head). Numbers on arrows represent the strength of temporal associations (standardised directed distances), with larger absolute values indicating stronger temporal relationships. (B) In- and out-strength centrality measures the total incoming connections or influence that a node has in a network, indicating whether changes precede those of other items, or whether changes follow those of other items.

Directed DTW analysis (Figure 3A) revealed that, within individuals, changes in SBP, DBP, MAP or PP over time were associated with subsequent inverse changes in IADL function (*P* < .05; red arrows). This means that a relative increase in SBP, DBP, MAP or PP between two time points (e.g. from T1 to T2) was associated with a decline in IADL function in the following interval (e.g. from T2 to T3). Conversely, relative decreases in these BP parameters were associated with subsequent improvements in IADL function. Additionally, changes in DBP over time were positively associated with subsequent changes in MMSE (*P* < .05; green arrow). This means that a relative decrease in DBP between two time points (e.g. from T1 to T2) was associated with a later decrease in MMSE in the following interval (e.g. from T2 to T3). Alternatively, increases in DBP were associated with later increases in MMSE. Finally, there was a positive temporal association between changes in ADL and IADL and subsequent changes in SBP (*P* < .05; green arrows), suggesting that changes in functional status may at times precede changes in SBP. All reported associations remained statistically significant after applying the Benjamini-Hochberg correction for multiple testing.

Sensitivity analyses were conducted to assess the robustness of the directed DTW findings (Supplementary Figure S4  A-D). The observed temporal associations remained consistent across all subgroups, indicating that the findings were robust regardless of sex, grip strength, antihypertensive use at baseline or changes in antihypertensive medication over time.

Figure 3B illustrates the in- and outstrength measures. DBP exhibited the highest relative out strength (*P* = .02), indicating that changes in DBP were most frequently followed by subsequent changes in ADL, IADL and MMSE. Conversely, IADL exhibited the highest in strength (*P* = .05), indicating that IADL most frequently exhibited changes after changes in BP measurements.

## Discussion

In this 5-year prospective study of 85-year-olds, we found that changes in BP over time were associated with later changes in functional status and cognitive function. Specifically, changes in any BP measure over time were associated with inverse changes in IADL function whereas changes in DBP were associated with changes in the same direction in cognitive function. These findings highlight the complex dynamics between BP and functional status and cognitive function in very old age.

The interpretation of our findings requires careful consideration, particularly because most prior studies have focused on absolute BP values, whereas our approach examined within-person trajectories over time. This distinction complicates direct comparisons to previous research, as individuals with similar relative changes may have completely different absolute BP values.

Our finding that changes in BP over time were associated with inverse changes in IADL function aligns with results of the DANTON trial [29]. This study assessed the effect of discontinuing antihypertensive treatment in older adults with dementia in nursing homes. Participants who discontinued antihypertensive treatment experienced an expected increase in SBP and DBP, but also a higher rate of serious adverse events. This possibly indicates that a relative increase in BP is harmful even in vulnerable older adults. Similarly, our observation that changes in DBP over time were associated with later changes in cognitive function aligns with findings by Qiu et al. [30], who reported greater BP declines in individuals who developed dementia compared to those who did not. Together, these studies suggest that changes in BP in either direction might be indicative of later functional or cognitive decline.

Several potential mechanisms may underlie the temporal associations we observed between BP changes and functional, and cognitive outcomes. Large fluctuations or sustained changes in BP over time may reflect impaired physiological buffering capacity, such as arterial stiffness or autonomic dysregulation [31, 32]. These instabilities may lead to oscillations in cerebral blood flow, potentially contributing to neuronal injury and cognitive decline [31]. Alternatively, our finding that declining DBP preceded cognitive decline might also reflect reverse causation, where early neurodegenerative processes might impair BP regulation before cognitive symptoms become clinically apparent. Frailty may further modify these associations: previous studies have shown that the effects of high BP may differ depending on frailty status [4, 33, 34]. Our sample consisted of relatively fit older adults, and care should be taken in generalising these results to populations with frailty.

Clinically, our findings underscore the importance of careful and individualised BP management in adults aged 85 years and older. Rather than targeting specific absolute BP thresholds, these results suggest that maintaining stability in BP over time may be more important than achieving reductions or tolerating fluctuations. Both increases and decreases in BP appeared to precede negative outcomes, perhaps indicating a U-shaped relationship between BP and health in this age group, where deviation in either direction can be detrimental.

This study has several notable strengths. Most importantly, it applies a novel analytical approach, serving as a proof of principle for capturing temporal dynamics in complex longitudinal data. By aligning individual trajectories before aggregating effects, DTW allows us to identify within-person changes and directional relationships that may not be identified in more traditional longitudinal analyses. Furthermore, we used the data of the Leiden 85-plus study, a well-characterised cohort with repeated measurements over a 6-year period.

However, some important limitations must also be acknowledged. First, the study is observational in nature, which introduces the possibility of residual confounding. For example, antihypertensive treatment may have been tapered in participants with deteriorating health, potentially leading to increases in BP and concomitant functional decline, reflecting indication bias rather than a causal relationship. Second, selection bias may have occurred, as we included only participants who survived with at least two follow-up measurements. This may have led to the inclusion of relatively fitter individuals. This disparity is partly reflected when comparing included and excluded participants. Finally, DTW analyses are most robust with longer and more variable time series; with only three measurement points and low within-person variability for some variables, the detection of reliable temporal ordering may have been limited. Future studies should explore the use of DTW in other longitudinal datasets, ideally stratified by antihypertensive use and frailty status, to better understand the temporal dynamics in these subgroups.

In conclusion, this study demonstrates that changes in BP are temporally associated with subsequent changes in functional status and cognitive function in adults aged 85 years and older. Using a novel analytical approach, we found that both increases and decreases in BP, particularly in diastolic BP, can precede functional and cognitive decline. These findings suggest that stability in BP may be more important than absolute values in this age group and highlight the potential of DTW as a tool to uncover individual-level temporal dynamics in ageing research.

## Supplementary Material

afag006_Supplementary_materials

## Data Availability

Data available from the authors on request.
