# Supplementary material for: Temporal dynamics of blood pressure, functional status and cognitive function in adults aged 85 years and older: a dynamic time warping approach in the Leiden 85-plus study
Source: Age Ageing. 2026 Jan 29;55(1):afag006. doi: 10.1093/ageing/afag006 (PMC12853301; doi:10.1093/ageing/afag006)
Supplement: afag006_Supplementary_materials [file afag006_supplementary_materials.docx]

Supplementary file: *Temporal Dynamics of Blood Pressure, Functional Status and Cognitive Function in Adults Aged 85 Years and Older: A Dynamic Time Warping Approach in the Leiden 85-plus Study*

# Contents

- **Supplementary Table 1 Page 2**
- **Supplementary Figure 1 Page 3**
- **Supplementary Figure 2 Page 4**
- **Supplementary Figure 3 Page 5**
- **Supplementary Figure 4 Page 6**
- **STROBE Checklist Pages 7 – 10**

| **Supplementary table 1. Baseline characteristics and comparison of included and excluded participants (aged 85 years)** | | | | |
| --- | --- | --- | --- | --- |
| Characteristic | Entire population (n = 599) | Study sample (n = 429) | Excluded (n=170) | *p-*value |
| **Demographics** |  |  |  |  |
| Female, n (%) | 397 (66) | 296 (69) | 101 (59) | 0.03 |
| ≤ Primary education, n (%) | 387 (65) | 264 (62) | 123 (72) | 0.004 |
| Institutionalized, n (%) | 107 (18) | 62 (15) | 45 (27) | <0.001 |
| **Lifestyle Factors** |  |  |  |  |
| Ever Smoker, n (%) | 283 (47) | 194 (45) | 89 (52) | 0.10 |
| Regular alcohol use, n (%) | 295 (49) | 211 (49) | 84 (49) | 0.85 |
| **Medical History** |  |  |  |  |
| Stroke, n (%) | 61 (10) | 40 (9) | 21 (12) | 0.25 |
| Myocardial infarction, n (%) | 63 (11) | 38 (9) | 25 (15) | 0.31 |
| Diabetes Mellitus, n (%) | 86 (14) | 58 (14) | 28 (17) | 0.28 |
| Parkinson’s Disease, n (%) | 16 (3) | 10 (2) | 6 (4) | 0.39 |
| **Medication Use** |  |  |  |  |
| Anti-hypertensive medication, n (%) | 224 (37) | 166 (39) | 58 (34) | 0.68 |
| **BP Metrics** |  |  |  |  |
| SBP, mmHg, mean (SD) | 155 (19) | 156 (18) | 152 (19) | 0.01 |
| DBP, mmHg, Mean (SD) | 77 (10) | 78 (9) | 74 (10) | <0.001 |
| MAP, mmHg, mean (SD) | 103 (11) | 104 (11) | 100 (11) | <0.001 |
| PP, mmHg, Mean (SD) | 78 (15) | 79 (15) | 78 (17) | 0.47 |
| **Functional and Cognitive status** | |  |  |  |
| ADL, median (IQR) | 10 (9 - 15) | 10 (9 - 13) | 11 (9 - 18) | <0.001 |
| IADL, median (IQR) | 18 (12 - 27) | 17 (12 - 24) | 23 (15 - 33) | <0.001 |
| MMSE, median (IQR) | 26 (22 - 28) | 26 (24 - 28) | 24 (18 - 28) | <0.001 |
| Abbreviations: SD = Standard deviation; IQR = Interquartile range | | | | |


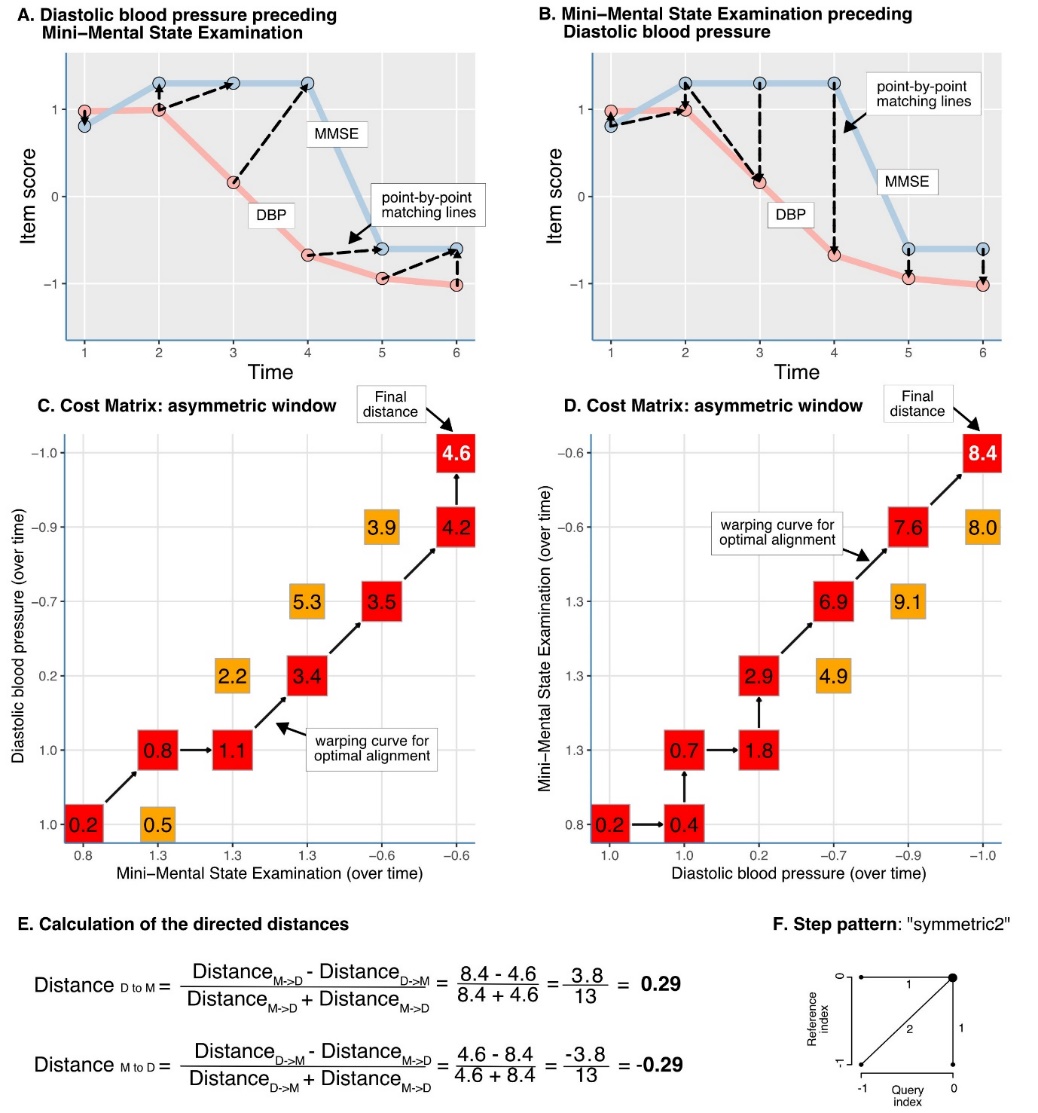


**Supplemental Figure 1** Example of directed DTW analysis tracking temporal variations between two variables in a single participant. Panels A and B show score trajectories for Diastolic Blood Pressure (DBP) and MMSE over six time points. Black dotted arrows illustrate the warping process, which aligns one variable's trajectory with another within time constraints. Panels C and D show Local Cost Matrices (LCM) used by the DTW algorithm to find optimal alignment. The analysis starts at bottom-left (LCM [1,1]) and proceeds to top-right (LCM [6,6]), selecting the path with minimum cumulative cost. Panel E shows the computed directed DTW distances. The DTW distance from DBP to MMSE is 4.6, while the reverse is 8.4, suggesting changes in DBP preceded changes in MMSE.


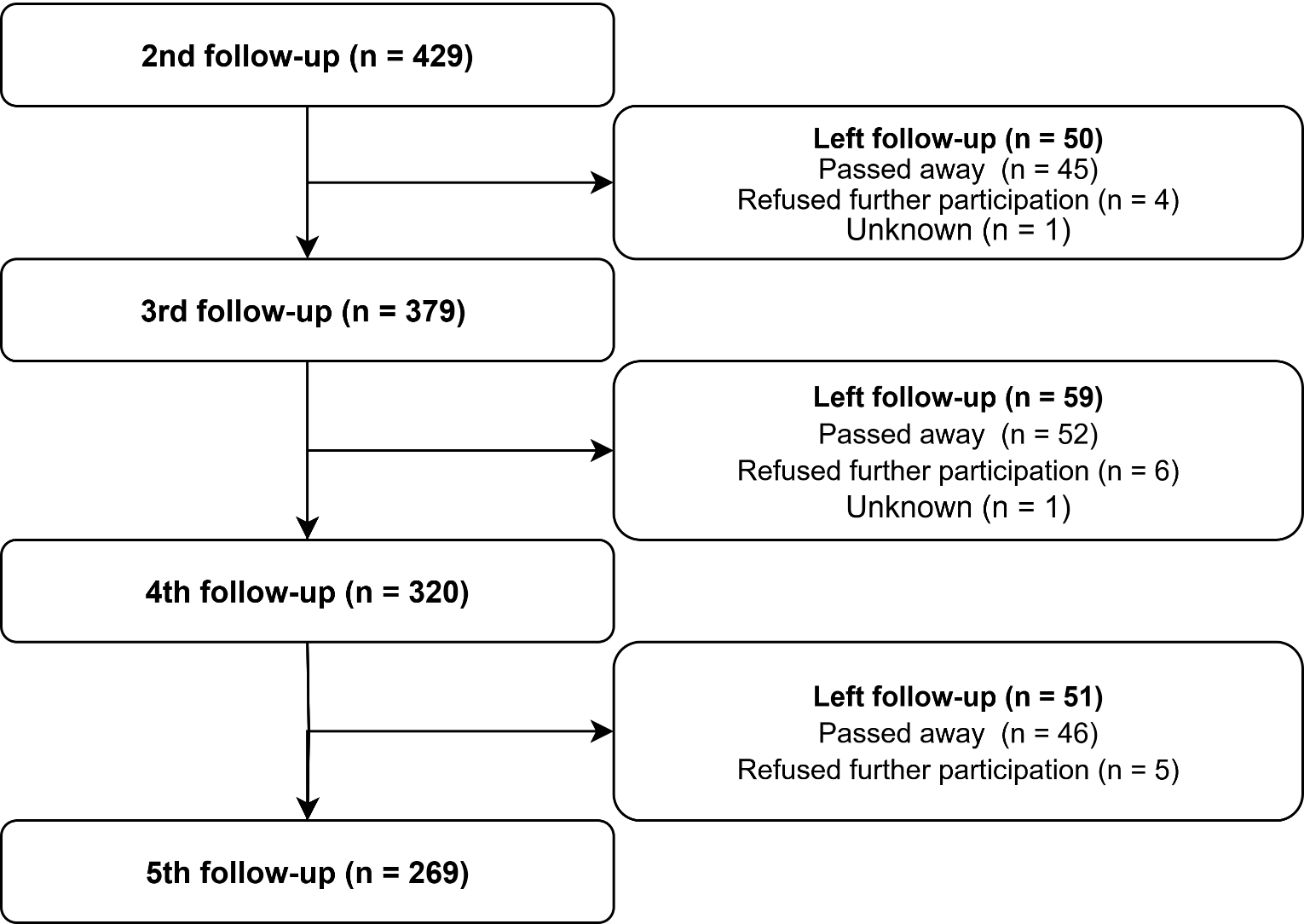


**Supplemental Figure 2**  Overview of follow-up duration distribution and reasons for loss of follow-up within the analytical sample.


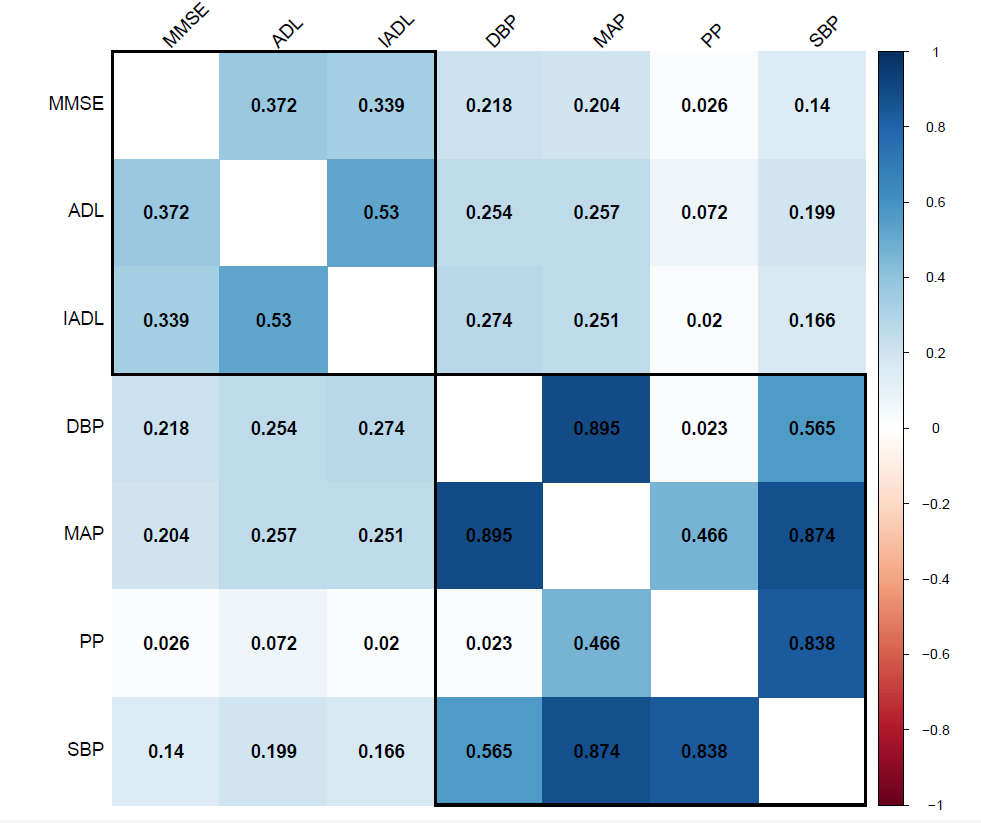


**Supplemental figure 3**: The heatmap displays the correlation coefficients between blood pressure parameters (systolic blood pressure, diastolic blood pressure, mean arterial pressure [MAP], and pulse pressure [PP]) and functional measures (ADL, IADL, and MMSE) across all participants at a single timepoint. A positive correlation means that higher values in one variable tend to be associated with higher values in the other variable at the same timepoint. Cooler colors indicate stronger positive correlations, while warmer colors represent negative correlations.

| 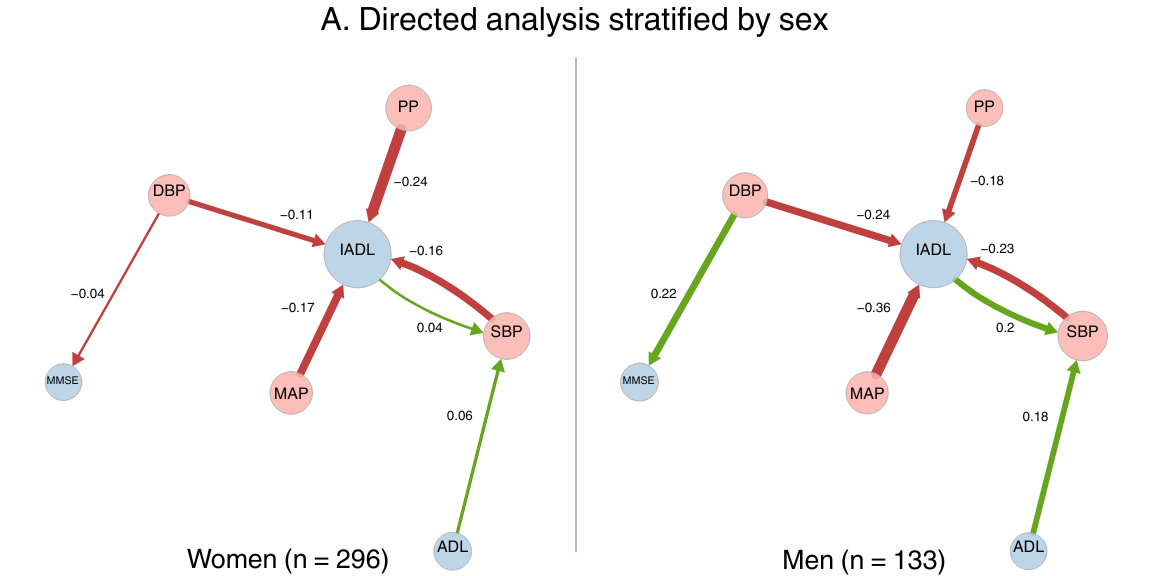 | 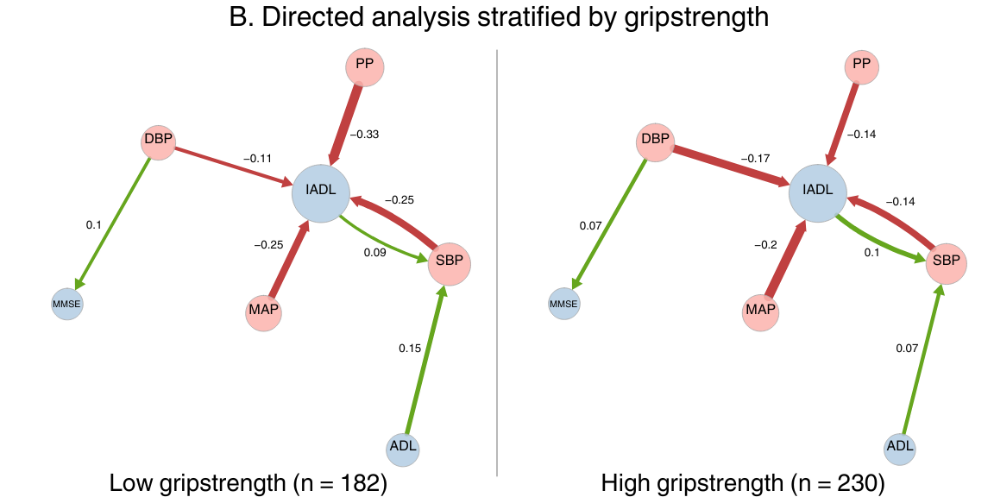 |
| --- | --- |
| 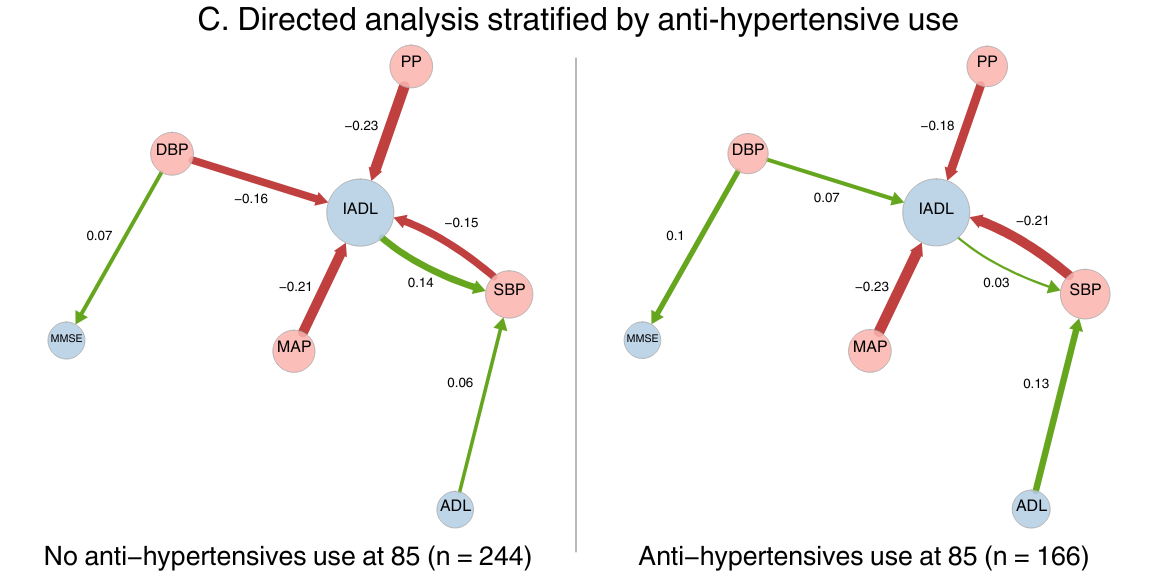 | 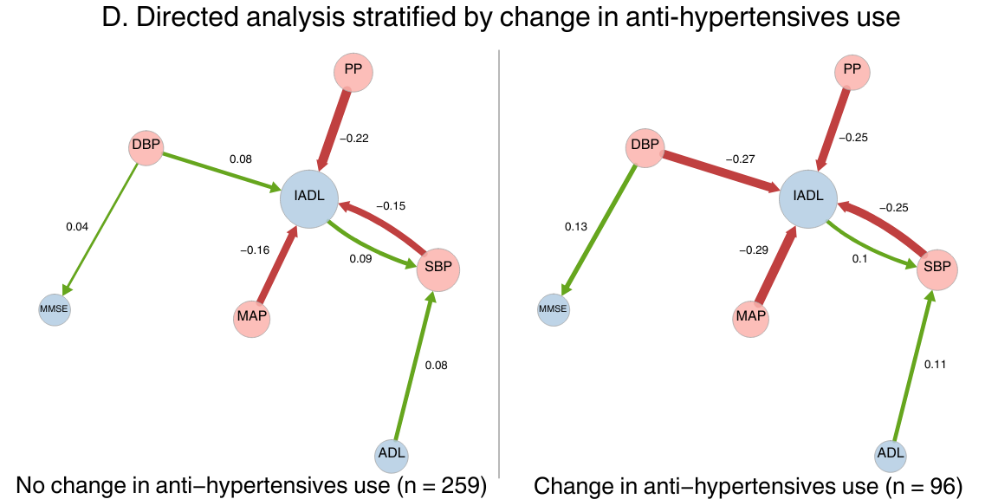 |

**Supplemental Figure 4:** Sensitivity analyses of directed DTW analysis stratified by participant characteristics. Shows time-lagged relationships between blood pressure parameters (SBP, DBP, MAP, PP) and functional measures (MMSE, ADL, IADL) for subgroups: (A) Sex (male vs. female), (B) Grip strength (below median vs. median and higher, adjusted for sex), (C) Antihypertensive use at baseline (yes vs. no), (D) Changes in antihypertensive use during follow-up (no change vs. change). Arrows indicate significant temporal associations (p < 0.05), consistent with the main analysis shown in Figure 3A.

STROBE Statement—Checklist of items that should be included in reports of ***cohort studies***

|  | Item No | Recommendation | Location |
| --- | --- | --- | --- |
| **Title and abstract** | 1 | (*a*) Indicate the study’s design with a commonly used term in the title or the abstract |  |
|  |  | (*b*) Provide in the abstract an informative and balanced summary of what was done and what was found | A. Title page  B. Abstract (page 2) |
| Introduction | | | |
| Background/rationale | 2 | Explain the scientific background and rationale for the investigation being reported | Introduction |
| Objectives | 3 | State specific objectives, including any prespecified hypotheses | End of introduction |
| Methods | | | |
| Study design | 4 | Present key elements of study design early in the paper | Methods |
| Setting | 5 | Describe the setting, locations, and relevant dates, including periods of recruitment, exposure, follow-up, and data collection | Methods |
| Participants | 6 | (*a*) Give the eligibility criteria, and the sources and methods of selection of participants. Describe methods of follow-up | A. Methods  B. NA |
|  |  | (*b*) For matched studies, give matching criteria and number of exposed and unexposed |  |
| Variables | 7 | Clearly define all outcomes, exposures, predictors, potential confounders, and effect modifiers. Give diagnostic criteria, if applicable | Methods |
| Data sources/ measurement | 8* | For each variable of interest, give sources of data and details of methods of assessment (measurement). Describe comparability of assessment methods if there is more than one group | Methods |
| Bias | 9 | Describe any efforts to address potential sources of bias | Methods, sensitivity analysis  Discussion  Supplementary table 1 |
| Study size | 10 | Explain how the study size was arrived at | Methods paragraph 1-2 |
| Quantitative variables | 11 | Explain how quantitative variables were handled in the analyses. If applicable, describe which groupings were chosen and why | Methods – statistical analysis |
| Statistical methods | 12 | (*a*) Describe all statistical methods, including those used to control for confounding | Methods – statistical analysis |
|  |  | (*b*) Describe any methods used to examine subgroups and interactions |  |
|  |  | (*c*) Explain how missing data were addressed |  |
|  |  | (*d*) If applicable, explain how loss to follow-up was addressed |  |
|  |  | (*e*) Describe any sensitivity analyses |  |
| Results | | |  |
| Participants | 13* | (a) Report numbers of individuals at each stage of study—eg numbers potentially eligible, examined for eligibility, confirmed eligible, included in the study, completing follow-up, and analysed | Figure 1  Results paragraph 1 |
|  |  | (b) Give reasons for non-participation at each stage |  |
|  |  | (c) Consider use of a flow diagram |  |
| Descriptive data | 14* | (a) Give characteristics of study participants (eg demographic, clinical, social) and information on exposures and potential confounders | A. Results paragraph 1  Table 1  B: NA (only participants without missing data included in analysis)  C  Results paragraph 1 Supplemental figure 2 |
|  |  | (b) Indicate number of participants with missing data for each variable of interest |  |
|  |  | (c) Summarise follow-up time (eg, average and total amount) |  |
| Outcome data | 15* | Report numbers of outcome events or summary measures over time | Results paragraph 3 |

| Main results | 16 | (*a*) Give unadjusted estimates and, if applicable, confounder-adjusted estimates and their precision (eg, 95% confidence interval). Make clear which confounders were adjusted for and why they were included | Results paragraph 4 |
| --- | --- | --- | --- |
|  |  | (*b*) Report category boundaries when continuous variables were categorized |  |
|  |  | (*c*) If relevant, consider translating estimates of relative risk into absolute risk for a meaningful time period |  |
| Other analyses | 17 | Report other analyses done—eg analyses of subgroups and interactions, and sensitivity analyses | Results paragraph 5-6 |
| Discussion | | | |
| Key results | 18 | Summarise key results with reference to study objectives | Discussion paragraph 2 |
| Limitations | 19 | Discuss limitations of the study, taking into account sources of potential bias or imprecision. Discuss both direction and magnitude of any potential bias | Discussion paragraph 7 |
| Interpretation | 20 | Give a cautious overall interpretation of results considering objectives, limitations, multiplicity of analyses, results from similar studies, and other relevant evidence | Discussion paragraph 4 |
| Generalisability | 21 | Discuss the generalisability (external validity) of the study results | Discussion paragraph 4 - 5 |
| Other information | | | |
| Funding | 22 | Give the source of funding and the role of the funders for the present study and, if applicable, for the original study on which the present article is based | Funding statement |

*Give information separately for exposed and unexposed groups.

**Note:** An Explanation and Elaboration article discusses each checklist item and gives methodological background and published examples of transparent reporting. The STROBE checklist is best used in conjunction with this article (freely available on the Web sites of PLoS Medicine at http://www.plosmedicine.org/, Annals of Internal Medicine at http://www.annals.org/, and Epidemiology at http://www.epidem.com/). Information on the STROBE Initiative is available at http://www.strobe-statement.org.
